# Environmental Preferences and Concerns of Recreational Road Runners

**DOI:** 10.3390/ijerph18126268

**Published:** 2021-06-10

**Authors:** Nadine Schuurman, Leah Rosenkrantz, Scott A. Lear

**Affiliations:** 1 Faculty of Environment, Simon Fraser University, 8888 University Drive, Burnaby, BC V5A 1S6, Canada; lrosenkr@sfu.ca; 2 Faculty of Health Sciences, Simon Fraser University, 8888 University Drive, Burnaby, BC V5A 1S6, Canada; salear@sfu.ca

**Keywords:** road running, built urban environment, online survey, environmental preferences, runnability

## Abstract

Recreational road running is growing in popularity and has been linked to numerous mental and physical health benefits. However, we know little about what environmental preferences or concerns runners have regarding participation in the sport, and whether differences exist across age and gender. We conducted a cross-sectional survey on recreational road runners to investigate the type of built and natural environments road runners prefer, as well as the safety and health concerns that may affect runners’ choice of environment. Responses were analyzed by age and gender. A total of 1228 road runners responded to the survey; 59.6% of respondents were women and 32.1% of respondents were men. Most respondents preferred to run on asphalt or sidewalk surfaces, and preferred well-lit, tree-lined routes. Major concerns for both men and women include animals and dangerous road conditions. Men and women differed significantly in their responses to the importance of running around others and their primary concerns while running. Results of this study serve to deepen our understanding of recreational road runners’ environmental preferences and concerns, providing valuable information for public health officials and city planners alike. This information must be considered if we are to continue to encourage uptake of running as a sport and reap its health effects.

## 1. Introduction

There are hundreds of millions of recreational runners worldwide [1]. Adding to this number, the sport has most recently seen a boon of new participants during the COVID-19 pandemic as gyms and indoor classes have closed down [2,3,4]. In April 2020, “Runkeeper”—a fitness tracking app—reported a 667% increase in registrations and a 105% increase in monthly users compared to the same time the year before [3]. Many newcomers to the sport are hopeful to continue their running routines once pandemic measures have been lifted [3].

Running is linked to numerous health benefits, both physical and psychological. Studies have suggested that runners have a 25–40% reduced risk of premature mortality, as well as significantly lower rates of cardiovascular disease and certain cancers [5,6,7,8,9]. In terms of psychological benefits, studies have shown that running can improve both mood and mental health, especially depression and anxiety disorders [10,11,12].

Given the popularity of recreational running worldwide, and the numerous health benefits it accords, it is surprising to find there is a lacuna of information on runners’ environmental preferences and concerns, and how these may differ by age and gender. The idea that certain environments can be more or less runnable is slowly beginning to be examined more critically [13,14,15,16,17,18,19]. The few studies that have looked into this have largely focused on the perceived and actual benefits of certain running environments over others, such as restorative capacity, stress relief, or satisfaction [13,16,18]. However, more specific runner preferences with regard to the built environment remain understudied, such as the types of surfaces runners prefer to run on, or which features of the built environment they find conducive to running [18].

While very much different than running, we can glean from the walkability literature that knowledge of such preferences is important information, and that they do indeed differ by age and gender [20,21,22,23]. The availability and accessibility of preferred urban forms can dramatically influence walking behavior and uptake in the activity [24,25,26]. The body of knowledge that has emerged from these studies on walking has proven immensely helpful to city planners and policymakers alike, who increasingly recognize the importance of the built environment on promoting active lifestyles for all ages and genders [27].

In addition to their preferences, understanding runners’ environmental concerns is also important to designing neighborhoods and parks that encourage physical activity. Qualitative studies by Krenichyn (2006), Wesley and Gaarder (2004), and Clark (2013) suggest that there is a gendered component to some of these concerns, especially those around personal safety—all three studies described how women often felt unsafe while running in different urban environments [15,28,29]. While the relationship between age and these concerns are less well-researched, studies of other physical activities suggest that it, too, has an impact on a participants’ concerns [21,30].

In light of these omissions in the running literature, we designed a study that further explores the idea of an environment’s “runnability” by investigating the environmental preferences and concerns of recreational runners according to their age and gender. As this is the first paper of its kind, we focus broadly on two sub-objectives: (1) to understand the type of built and natural environments road runners prefer, and (2) to understand safety and health concerns that may affect runners’ choice of environment. Both sub-objectives also take into account age and gender. In doing so, we hope to achieve a better awareness of the types of routes that both impede and encourage people to run.

## 2. Materials and Methods

### 2.1. Study Design

This study was based on a cross-sectional survey. Two studies were conducted in parallel using one survey. The first sought to understand the environmental preferences of recreational road runners, and the second to understand the environmental preferences of recreational trail runners (forthcoming manuscript). We describe methods for the first study on road runners only.

### 2.2. Study Population and Recruitment

**Figure 1:**
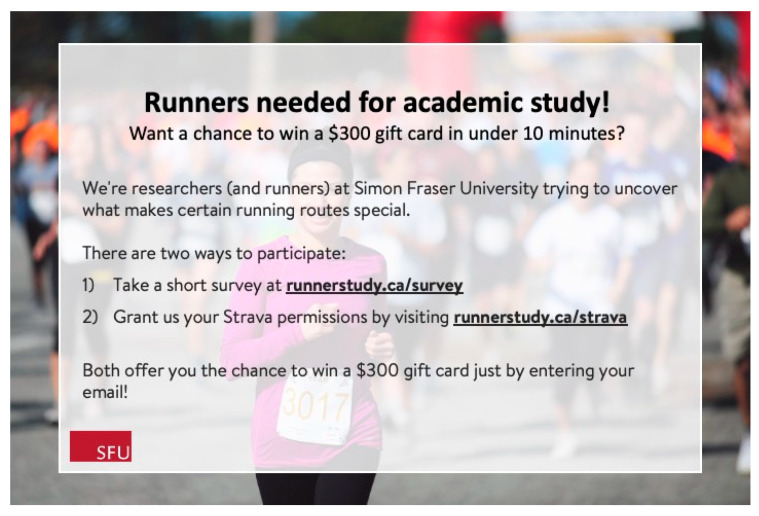
Runner study advertisement. Advertisement created for study recruitment purposes. The survey was advertised in tandem with another running-related research project being conducted using Strava permissions.

The study population consisted of a sample of people who self-identify as runners. No constraints were established concerning the gender, ethnicity, country of residence, or ability of the respondent; however, for ethics purposes, we limited the survey to only those aged 19 years and older. Both convenience and snowball sampling were used to recruit potential respondents from this population. Recruitment primarily took place through advertisements placed in North American running magazines and websites such as *Runner’s World*, *Canadian Running*, *Ultrarunning Magazine*, *Trail Runner Magazine*, and irunfar.com, as well as targeted social media ads on Facebook and Instagram. We also conducted in-person recruitment through local run clubs and running apparel stores using post-card advertisements (Figure 1). In several cases, we reached out by email to clubs and stores further afield. The survey was incentivized with the promise of a chance to win one of three CDN 300 gift cards from a running store.

### 2.3. Survey Development and Administration

An initial review of the literature was conducted to inform the development of the survey. However, since this review returned so few studies on the concept of runners’ environmental preferences—as differentiated from that of walkers or cyclists—survey development largely proceeded from the running experiences of the authors of this paper.

After initial development of the survey, questions were pretested for validity by four respondents not involved in development. All four of these people had numerous years of running experience and currently identified as a runner. The survey was modified in accordance with their feedback to ensure ease of interpretation and improved response rates.

Runners who were interested in participating in the study were directed to fill out our study’s survey, hosted on the SurveyMonkey platform online. Those who self-identified as road runners were directed to fill out 10 questions, separate from those who self-identified as a trail runner. The road-running survey included a variety of multiple choice and Likert scale questions (Table S1). Questions can be broadly classed under three categories: the respondents’ running profile (including age, gender, and running routines), their environmental preferences for running, and their environmental concerns for running (see Appendix A). Respondents were given between 17 January 2020 and 31 May 2020 to fill out the survey. Respondents were only allowed to fill in the survey once. Once submitted, they could no longer go back and change their responses.

Written informed consent was sought prior to participation in the survey. Approval for this study (Ethics Approval #2019s0322) was granted by Simon Fraser University’s Research Ethics Board.

### 2.4. Data Analysis

Data were analyzed through both statistical and qualitative analysis. Descriptive analysis was conducted on all survey answers using Excel, V.16.45. Chi-square tests of independence were also performed using SPSS, V.27 to assess whether meaningful differences between genders existed in respondent’s answers to survey questions. For the two questions that permitted multiple responses, all possible combinations of answers were compared to ensure that requirement for independence of observations was met (for example, with regard to the question on concerns while running, we treated those who selected distracted drivers or fear of other people as separate answers from those who selected both options). However, due to several cells having expected counts less than five, a Chi-square test could not be completed for the question pertaining to features of the built environment conducive to running.

For the same reason (i.e., low expected counts), we could not conduct chi-square tests of independence to assess the presence of meaningful differences across age groups by question. We thus opted to present these results descriptively only.

Thematic and content analysis were performed using NVivo, version 12.6.0 for questions that allowed free text responses when selecting “other” as an answer. Responses to free text were also coded by gender to determine if patterns exist between men and women. There were not enough free text responses by those from each age group to conduct a meaningful qualitative analysis for responses by age.

## 3. Results

One thousand two hundred and twenty-eight people responded to the survey, self-identifying as a road runner. Respondents were included in the analysis so long as they answered at least one question. Table 1 illustrates the breakdown of responses to each question by gender and provides the results of chi-square tests of independence identifying whether respondents’ answers differed significantly by gender. Table A1 (Appendix A) illustrates the breakdown of responses to each question by age. Response rates varied by question, ranging from 85.0% to 100.0%. Seven-hundred and seventy-eight respondents answered all of the questions.

### 3.1. Runner Profile

Seven hundred and thirty-two respondents (59.6%) identified as a woman, 394 respondents (32.1%) identified as a man, and six respondents (0.5%) identified as other, for a total of 1132 respondents. Ninety-six respondents (7.8%) did not complete this question. The age breakdown of respondents is as follows: 292 respondents (23.8%) identified as 19–24 years old, 269 (21.9%) respondents identified as 25–34 years old, 236 respondents (19.2%) identified as 35–44 years old, 206 respondents (16.8%) identified as 45–54 years old, 106 respondents (8.6%) identified as 55–64 years old, and 22 respondents (1.8%) identified as 65+ years old. Ninety-seven respondents (7.9%) did not complete this question.

Respondents have been running on average for 10.3 years (range = 0.25–58 years). Just over half (50.8%) of respondents have been running for less than seven years. For women, this average is 9.6 years (range: 1–46 years), while for men, it is 11.6 years (range: 0.25–58 years). There was a significant association between gender and number of years respondents have been running (*X*^2^ (6, *N* = 1044) = 15.986, *p* = 0.014), with a medium effect size (*V* = 0.124).

By age group, those 19–24 have been running for 5.0 years on average (range: 0.25–17 years), those 25–34 have been running for 7.8 years on average (range: 1–25 years), those 35–44 have been running for 11.1 years on average (range: 1–39 years), those 45–54 have been running for 15.3 years on average (range: 1–40 years), those 55–64 have been running for 18.8 years on average (range: 2–46 years), and, finally, those 65+ have been running for 25.6 years on average (range: 6–58 years).

Respondents run on average 3.9 days/week. For women, this average decreased to 3.75 days/week and for men it increased to 4.21 days/week. There was a significant association between gender and number of days run per week (*X*^2^ (6, *N* = 1126) = 34.927, *p* = 0.014), with a medium effect size (*V* = 0.176). Exactly 50% of respondents run less than 30 km total a week. This percentage increases to 62.7% for women and decreases to 37.8% for men. There was a significant association between gender and distance run per week (*X*^2^ (6, *N* = 1126) = 90.961, *p* = 0.000), with a large effect size (*V* = 0.284).

By age group, those 19–24 run on average 4.0 days a week, those 25–34 run on average 3.7 days a week, those 35–44 run on average 3.8 days a week, those 45–54 run on average 3.8 days a week, those 55–64 run on average 4.1 days a week, and, finally, those 65+ run on average 3.7 days a week. Over 50% of respondents in the age groups 19–24, 25–34, 35–44, and 45–54 run less than 30 km per week, compared to only 41% of respondents aged 55–64 and 65+.

### 3.2. Environmental Preferences for Running

On a scale from 1 (not important) to 5 (very important), the average respondent’s answer to the importance of running around others was 2.26. When examined by gender, this average increased to 2.49 for women, and decreased to 1.86 for men. There was a significant association between gender and ranked importance (*X*^2^ (4, *N* = 1114) = 66.83, *p* = <0.001), with a large effect size (*V* = 0.245). When examined by age, the greatest proportion of respondents for each age group (with the exception of those 65+) responded that it was not very important to run with/around others. For those = 65+, the greatest proportion of respondents selected that it was somewhat important to run with/around others.

When it came to preferences for running surfaces, results varied widely by surface type, with most respondents seeking out asphalt/paved surfaces and sidewalks often or always (Table 1). No significant difference was seen across genders for the frequency with which they choose to run on asphalt or sidewalk surfaces. However, a significant difference was found for unpaved and track surfaces, though effect sizes for both were small (*X*^2^ (4, *N* = 1125) = 19.156, *p* = 0.001, *V* = 0.130 and *X*^2^ (4, *N* = 1124) = 10.158, *p* = 0.038, *V* = 0.095, respectively). When examined by age group, there were no deviations in trends from the population as a whole.

**Figure 2:**
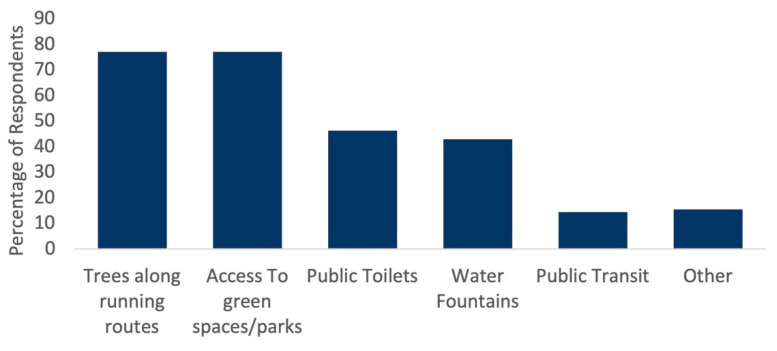
Percentage of respondents who find the following features of the urban environment conducive to running (n = 1228).

**Figure 3:**
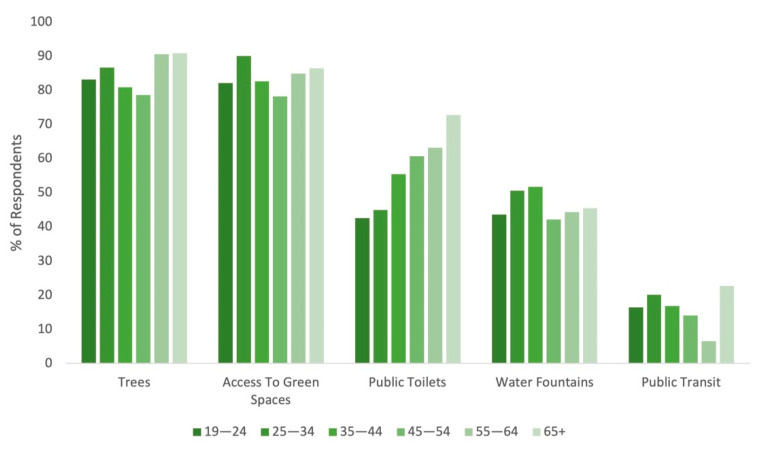
Percentage of respondents by age group who find the following features of the urban environment conducive to running (n = 1131).

Large and near-equivalent percentages of respondents found access to green spaces (77.1%) and tree-lined running routes (70.0%) to be the most conducive for running (Figure 2). Public toilets and water fountains were also highly valued by close to half of all respondents. For public toilets in particular, there was an increasing preference by age group, from 42.6% for those aged 19–24 to 72.7% for those aged 65+ (Figure 3). A total of 188 respondents provided their own answers. These results were analyzed thematically; twelve themes were identified. In order of most common to least common, they were: continuity of running path, quiet (away from traffic and people), street/path lighting, access to green/blue space, maintained paths, clearly defined pedestrian routes/safety features (e.g., crosswalks, separated pedestrian/cyclist paths), aesthetically pleasing, access to amenities, terrain specifics (e.g., hilly routes, flat routes, mix of both), wide running paths, clear sightlines and other people around for safety, and running path is nearby.

**Figure 4:**
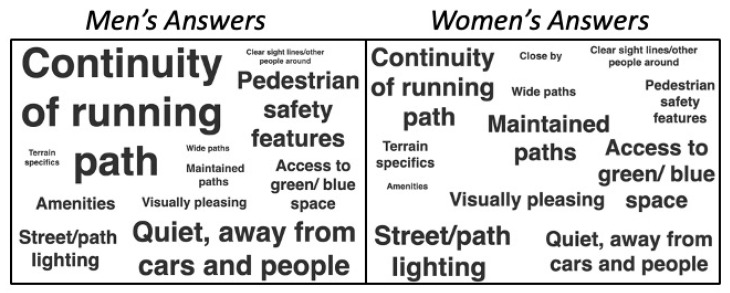
Thematic analysis: Features of the built environment conducive to running.

When examined by gender, men’s and women’s written answers showed several differences. Women tended to emphasize the need for well-lit street path lighting, and a few mentioned the merits of having good running routes “close-by”. Men, on the other hand, tended to emphasize the importance of having a continuous running path and quieter running routes. For men, not one mentioned the need for a good running route close-by. Figure 4 demonstrates the frequency of each theme by gender.

This figure illustrates the themes identified for the open-ended part of the question “what features of the built environment are most conducive to running?”. The size of the font for each theme corresponds to the frequency with which men’s and women’s responses aligned with that theme.

### 3.3. Concerns for Running

**Figure 5:**
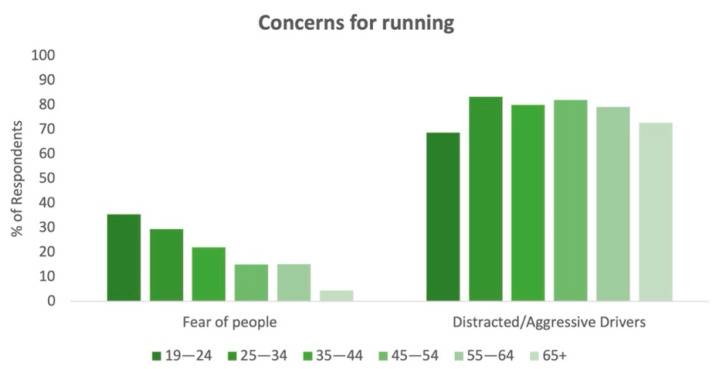
Concerns for running by age group.

Safety-wise, 61.0% of respondents were concerned about distracted or aggressive drivers on their runs; 20.4% of respondents feared other people while running. A chi-square test of respondents’ answers demonstrated a statistically significant difference between men and women (*X*^2^ (3, *N* = 1285) = 104.688, *p* = 0.000), with a large effect size (*V* = 0.285). In particular, women responded that they feared other people more while out on runs to a greater degree than men, and worried less about distracted drivers. Fear of people also demonstrated a decline as age increased, with those over 65 having the lowest percentage of respondents (4.5%) that responded with this as a concern (Figure 5).

A total of 18.6% of respondents wrote in their own response to this question for concerns they have while running. These responses were organized into eight themes. By far, the most common theme identified from the responses had to do with concerns about animals. This included both wild animals (such as deer, bears, snakes, etc.) and insects (bees and wasps), but also to a large degree leashed or unleashed dogs. Getting bitten or chased by a dog was mentioned by 90 respondents. “Dangerous road conditions” was another major theme. Respondents here referred to dangerous conditions due to acclimate weather (e.g., black ice on sidewalks, slippery paths), dangerous conditions due to poorly maintained infrastructure (e.g., potholes, uneven ground, construction sites), or both. Compared to these first two themes, the remaining six themes were mentioned less frequently but were still alluded to by several respondents. These include (from most frequent to least): worries about getting injured, distracted cyclists and pedestrians (e.g., people walking while on their phone), darkness, fear of being attacked or harassed by a stranger, unsafe road design (e.g., narrow shoulders, few crosswalks), and being remote. With regard to darkness, it seems respondents’ concerns were related to two major ideas: not being seen by drivers or cyclists, and, on the flip side, not being able to see any potential threats posed by other humans or animals. Similarly, with regard to being remote, respondents’ concerns here primarily revolved around not having others around if something were to go wrong, such as getting injured or being assaulted. Seventeen responses did not fit neatly into any of these themes and were classed as other. They included mentions of needles, sharps or other biohazards, farm equipment, and COVID-19, to name a few (our survey was conducted at the very beginning of the COVID-19 pandemic and ended only a month into mandated lockdowns).

**Figure 6:**
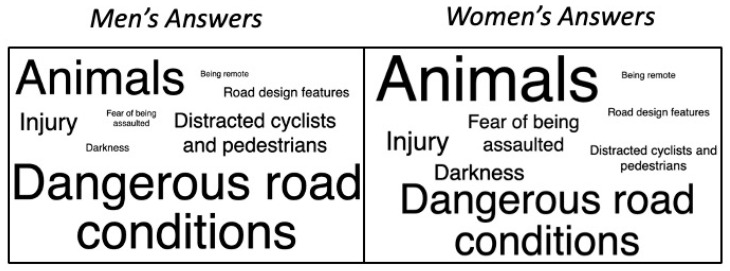
Thematic analysis: “Safety-wise, what are your primary concerns while running?”.

When examined by gender, men’s and women’s written answers showed several differences. Both animals and the fear of being assaulted are greater concerns for women than they are for men. On the other hand, men showed greater concern over distracted cyclists and pedestrians, as well as dangerous road conditions. Figure 6 demonstrates the frequency of each theme by gender.

This figure illustrates the themes identified for the open-ended part of the question “Safety-wise, what are your primary concerns while running?”. The size of the font for each theme corresponds to the frequency with which men’s and women’s responses aligned with that theme.

**Figure 7:**
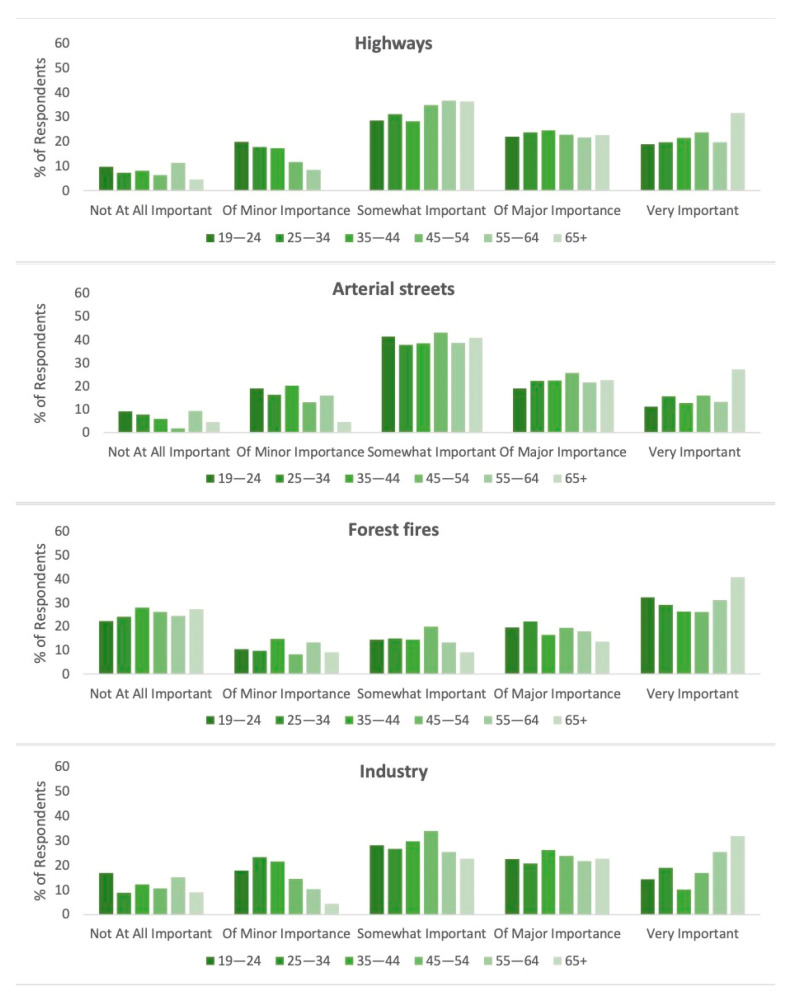
Importance of avoiding pollution from the following sources by age group.

Lastly, for the survey question regarding the importance of avoiding pollution from various sources, the largest number of respondents found it *somewhat* important to avoid pollution from highways, arterial streets, and industry. Results of chi-square tests for each of the pollution sources listed showed no significant association with gender. Broken down by age, however, we see that a greater percentage of those over 65 found it “very important” to avoid each listed source of pollution than any other age group (Figure 7). Open-ended responses were classified by content according to the pollution source referenced. They include burning garbage (2 respondents), dust (1 respondent), food processing plants (1 respondent), food truck generators (1 respondent), forest fires (1 respondent), garbage (smell and litter) (3 respondents), noise pollution (2 respondents), pesticides and agricultural pollution (3 respondents), second-hand smoke (1 respondent), and sewage (4 respondents). Eighteen responses did not address the question being asked and were therefore excluded from this classification.

## 4. Conclusions

In this study of environmental preferences and concerns of 1228 self-identified road runners, results show that an “ideal” running route (as selected by the majority of respondents) would take place on asphalt or sidewalk surfaces that are tree-lined and close to green spaces. These routes would preferably be well-lit, quiet, and have few intersections to disrupt the continuity of the run. They would also be well-maintained with dangers such as icy sidewalks or potholes minimized.

Our results also indicate that men and women are different in many ways when it comes to running. This includes runners’ characteristics such as age, running experience, and intensity of training, but also their environmental preferences and safety concerns. These gender differences need to be accounted for in how we go about planning communities that more equitably promote running for both men and women.

When it comes to environmental preferences, there is a small but significant difference in men’s vs. women’s surface choice. Men prefer to run on unpaved surfaces and running tracks more than women, though no significant difference was found for either asphalt or sidewalk surfaces (which both men and women favored overall). Understanding these preferences is important—both Deleen et al. (2019) and Ettema (2015) have pointed out that the “comfort” of running surfaces play a major role in both the frequency with which a runner runs and the perceived attractiveness and restorative capacity of the route [16,18].

In terms of features of the environment that runners find conducive to running, no significant difference was found between genders to the closed-ended responses. Trees along running routes and access to green space were both top answers for men and women, echoing other studies which have found that running in parks or on mostly green routes was significantly associated with the perceived attractiveness of the running environment [16,18].

In examining the open-ended responses to this question, though, there appears to be several discrepancies between genders. For example, men seem to place a greater value on the continuity of the running path and having pedestrian safety features in place, compared to women who, instead, appear to place a greater value on access to green and blue space and maintained paths. While we are unaware of any running-focused study that has examined gendered differences with respect to these features, studies from the walkability literature have done so. Findings from these studies, however, remain inconclusive, with both support for and against certain gender differences, depending on the study population [31,32,33]. Further research in this area on runners specifically is needed to validate our findings.

As for safety concerns, the divide between genders is even larger. In particular, women feared for their personal safety from other people a great deal more than men (26.7% for women compared to 6.8% for men). This is reflected in both their open- and closed-ended responses to the question on safety, but also in their responses to other questions. Adequate lighting and clear sightlines were mentioned by a greater percentage of women (14.0% and 3.7%, respectively) than men (10.0% and 1.2%, respectively). Women also felt it more important than men to run around others (22.5% of women said it was important or very important to run around others, compared to 8.8% of men). There are a few reasons why this may be, but based on several of the women’s responses, it seems clear that at least one explanation is the feeling of safety that comes with having a running partner or group. In her autoethnographic study of distance runners, Allen-Collinson (2008) found that running with company helped reduce feelings of discomfort and unsafety created by negative social interactions with others such as verbal or even physical harassment [34]. Viewed collectively, women’s responses indicate that many women are worried about dangers posed to them by others and take steps to either limit their running to safer times of day, to safer areas, or to when they can run with, or around, others. While further research is needed, it is possible that this influences the amount women run, as seen in the runner profile.

Though we could not conduct statistical analysis to determine whether respondents’ preferences and concerns were significantly different according to age, our descriptive reporting of the data suggest that certain age groups have particular preferences and concerns with regard to running compared to other age groups. Further study on these differences at a statistical level is required to determine whether this is an important factor to consider in planning healthy active communities.

Acknowledging how men and women of varying ages differ when it comes to their running preferences and concerns will have important implications for how we choose to plan and design communities and cities going forward. Responses of both men and women runners across the age spectrum show that what runners want is not always the same as what the literature tells us pedestrians want. In other words, we cannot just apply the same principles of walkability and hope it works for runners too. As running continues to grow in popularity, we must consider how we can create environments that better balance the needs of runners and pedestrians alike. Research in this area is just beginning, with researchers looking at what makes a city runnable [18], and attention being given to developing a novel “runnability” index [19]. Findings from our study will be an important contribution to future iterations of runnability indices.

Despite there being a deep literature on walkability [21,22,24,26,35,36], there is a deficit of literature on runnability. To the best of our knowledge, this study is the first of its kind to deal explicitly with the environmental variables (both natural and those related to the built environment) that contribute to runnability for men and women across the age spectrum. Moreover, it addresses the specific issues that runners themselves encounter with relation to the urban built environment. This paper fills a significant gap, though we do acknowledge several limitations. This study was broad in its scope and did not limit respondents based on their geography, allowing for a range of responses from both urban and rural areas. Though we view this as an asset of the study, our study would have benefited further if we had asked respondents to contextualize for us geographically where and when they typically run. First and foremost, geographical contextualization would have allowed us to split the inherent relationships between the data, allowing us to analyze and compare similar runners with each other according to their geography (e.g., the preferences of runners living in major urban centers could be parsed out from those of runners living in rural towns). As is, we are unable to determine how place influences runners’ preferences and concerns, and subsequently suggest this as an important area of future study. In the same vein, because our recruitment of runners targeted running groups and magazines mostly based in North America, it is likely that runners from this part of the world are overrepresented. While there is no literature that defines what a representative sample for recreational road runners is, we do recognize that our sample likely is skewed toward the North American running experience. It is possible that certain genders or ages are also over- or under-represented, though, again, it remains to be determined what representative means for this population.

It is important to note that this study also does not take into account what we deem the “convenience factor”. That is, although the survey focuses on eliciting runners’ preferences, we might in fact be getting what is convenient for that person, not what they would prefer if all options were accessible. For example, respondents that said they never seek out a track may live far away from one, and consequently prefer (or just do not have the time) to seek one out.

In conclusion, this study deepens our understanding of recreational road runners’ environmental preferences and concerns, and subsequently provides valuable information for public health officials and city planners alike. As running continues to grow in popularity, it will be important to develop built environments that meet the unique preferences and concerns of runners of all ages and genders and encourage greater participation in the sport.

## Figures and Tables

**Table 1:** Comparisons of runner characteristics, preferences, and concerns by gender.

Variable	Overall Sample n (%)	Men n (%)	Women n (%)	Chi-Square Tests of Independence
Age in Years				
19–24	290 (25.8)	90 (22.3)	200 (27.4)	*Χ*^2^ (5) = 9.386
25–34	268 (23.8)	87 (21.5)	181 (24.8)	*p* = 0.095
35–44	234 (20.8)	86 (21.3)	148 (20.3)	*V* = 0.091
45–54	206 (18.3)	75 (18.6)	131 (17.9)	*n* = 1124
55–64	104 (9.3)	44 (10.9)	60 (8.2)	
65+	22 (2.0)	12 (3.0)	10 (1.4)	
Missing or Other Gender (n = 104)				
No. of Years Running				
0–5	404 (38.7)	133 (36.3)	271 (40.0)	*Χ*^2^ (6) = 15.986
6–10	305 (29.2)	98 (26.8)	207 (30.5)	*p* = 0.014
11–15	130 (12.5)	51 (13.9)	79 (11.7)	*V* = 0.124 **
16–20	85 (8.1)	33 (9.0)	52 (7.7)	*n* = 1044
21–25	34 (3.3)	16 (4.4)	18 (2.7)	
26–30	41 (3.9)	10 (2.7)	21 (4.6)	
30+	45 (4.3)	25 (6.8)	20 (2.9)	
Missing or Other Gender (n = 194)				
Km Distance/Week				
<10	103 (9.1)	18 (4.6)	85 (11.6)	*Χ*^2^ (6) = 90.961
10–20	257 (22.8)	62 (15.7)	195 (26.6)	*p* = 0.000
21–30	248 (22.0)	69 (17.5)	179 (24.5)	*V* = 0.284 ***
31–40	181 (16.1)	70 (17.8)	111 (15.2)	*n* = 1126
41–50	176 (15.6)	76 (19.3)	100 (13.7)	
51–60	59 (5.2)	32 (8.1)	27 (3.7)	
60+	102 (9.1)	67 (17.0)	35 (4.8)	
Missing or Other Gender (n = 102)				
No. of Days Running/Week				
1	34 (3.0)	10 (2.5)	24 (3.3)	*Χ*^2^ (6) = 34.927
2	113 (10.0)	34 (8.6)	79 (10.8)	*p* = 0.000
3	356 (31.6)	99 (25.1)	257 (35.1)	*V* = 0.176 **
4	260 (23.1)	90 (22.8)	170 (23.2)	*n* = 1126
5	192 (17.1)	72 (18.3)	120 (16.4)	
6	126 (11.2)	61 (15.5)	65 (8.7)	
7	45 (4.0)	28 (7.1)	17 (2.3)	
Missing or Other Gender (n = 102)				
Importance of Running around Others				
1 (Not important)				
2	427 (38.3)	197 (50.6)	230 (31.7)	*Χ*^2^ (4) = 66.825
3	222 (19.9)	91 (23.4)	131 (18.1)	*p* = 0.000
4	268 (24.1)	67 (17.2)	201 (27.7)	*ϕ* = 0.245 ***
5 (Very important)	133 (11.9)	26 (6.7)	107 (14.8)	*n* = 1114
Missing or Other Gender (n = 114)	64 (5.7)	8 (2.1)	56 (7.7)	
Prefers to Run on Sidewalk				
Never	26 (2.3)	13 (3.3)	13 (1.8)	*Χ*^2^ (4) = 7.697
Rarely	127 (11.3)	53 (13.5)	74 (10.1)	*p* = 0.103
Sometimes	235 (20.9)	84 (21.4)	151 (20.7)	*V* = 0.083
Often	566 (50.4)	193 (48.6)	373 (51.0)	*n* = 1124
Always	170 (15.1)	50 (12.7)	120 (16.4)	
Missing or Other Gender (n = 104)				
Prefers to Run on Asphalt/Paved				
Never	10 (0.9)	2 (0.5)	8 (1.1)	*Χ*^2^ (4) = 3.654
Rarely	42 (3.7)	15 (3.8)	27 (3.7)	*p* = 0.455
Sometimes	184 (16.4)	61 (15.6)	123 (16.8)	*V* = 0.057
Often	682 (60.7)	232 (59.2)	450 (61.5)	*n* = 1124
Always	206 (18.3)	82 (20.9)	124 (16.9)	
Missing or Other Gender (n = 104)				
Prefers to Run on Unpaved				
Never	49 (4.4)	22 (5.6)	27 (3.7)	*Χ*^2^ (4) = 19.156
Rarely	228 (20.3)	61 (15.5)	167 (22.8)	*p* = 0.001
Sometimes	452 (40.2)	145 (36.8)	307 (42.0)	*V* = 0.130 *
Often	333 (29.6)	138 (35.0)	195 (26.7)	*n* = 1125
Always	63 (5.6)	28 (7.1)	35 (4.8)	
Missing or Other Gender (n = 103)				
Prefers to Run on Track				
Never	189 (16.8)	53 (13.5)	136 (18.6)	*Χ*^2^ (4) = 10.158
Rarely	380 (33.8)	131 (33.2)	249 (34.1)	*p* = 0.038
Sometimes	320 (28.5)	112 (28.4)	208 (28.5)	*V* = 0.095 *
Often	195 (17.3)	78 (19.8)	117 (16.0)	*n* = 1124
Always	40 (3.6)	20 (5.1)	20 (2.7)	
Missing or Other Gender (n = 104)				
Features of the Environment Conducive to Running (more than one response permitted)				
Trees along route	936 (28.2)	321 (27.1)	615 (28.8)	*NA*
Access to green spaces/parks	940 (28.3)	329 (27.7)	611 (28.6)	
Public toilets	561 (16.9)	192 (16.1)	369 (17.3)	
Water fountains	523 (15.7)	200 (16.9)	323 (15.1)	
Access to public transit	175 (5.3)	66 (5.6)	109 (5.1)	
Other	187 (5.6)	78 (6.6)	109 (5.1)	
Missing or Other Gender (n = 102)				
Concerns for Running (more than one response permitted)				
Distracted drivers	879 (61.0)	334 (73.2)	545 (55.3)	*Χ*^2^ (3) = 104.688
Fear of people	294 (20.4)	31 (6.8)	263 (26.7)	*p* = 0.000
Other	268 (18.6)	91 (20.0)	177 (18.0)	*V* = 0.285 ***
Missing or Other Gender (n = 102)				*n* = 1285
Importance of Avoiding Highway Pollution				
Not at all important	85 (7.6)	40 (10.2)	45 (6.2)	*Χ*^2^ (4) = 7.823
Of minor importance	181 (16.1)	60 (15.3)	121 (16.6)	*p* = 0.098
Somewhat important	386 (34.4)	118 (30.1)	238 (32.6)	*V* = 0.084 *
Of major importance	252 (22.5)	94 (24.0)	158 (21.6)	*n* = 1121
Very important	247 (22.0)	79 (20.2)	168 (23.0)	
Missing or Other Gender (n = 107)				
Importance of Avoiding Traffic Pollution from Arterial Streets				
Not at all important	73 (6.5)	31 (7.9)	42 (5.7)	*Χ*^2^ (4) = 5.777
Of minor importance	195 (17.3)	75 (19.1)	120 (16.4)	*p* = 0.216
Somewhat important	447 (39.8)	140 (35.7)	307 (41.9)	*V* = 0.072
Of major importance	247 (22.0)	86 (21.9)	161 (22.0)	*n* = 1124
Very important	162 (14.4)	60 (15.3)	102 (13.9)	
Missing or Other Gender (n = 104)				
Importance of Avoiding Forest Fire Pollution				
Not at all important	266 (23.7)	106 (27.0)	160 (21.9)	*Χ*^2^ (4) = 8.620
Of minor importance	122 (10.9)	46 (11.7)	76 (10.4)	*p* = 0.071
Somewhat important	172 (15.3)	67 (17.0)	105 (14.4)	*V* = 0.088
Of major importance	220 (19.6)	67 (17.0)	153 (20.9)	*n* = 1124
Very important	344 (30.6)	107 (27.2)	237 (32.4)	
Missing or Other Gender (n = 104)				
Importance of Avoiding Industry Pollution				
Not at all important	130 (11.6)	51 (13.1)	79 (10.8)	*Χ*^2^ (4) = 2.684
Of minor importance	213 (19.0)	76 (19.5)	137 (18.8)	*p* = 0.612
Somewhat important	327 (29.2)	110 (28.2)	217 (29.8)	*V* = 0.049
Of major importance	251 (22.4)	91 (23.3)	160 (21.9)	*n* = 1119
Very important	198 (17.7)	62 (15.9)	136 (18.7)	
Missing or Other Gender (n = 109)				

*V* = effect size (Cramer’s V). * A significant difference with a “small” effect size as per Cohen’s definition. ** A significant difference with a “medium” effect size as per Cohen’s definition. *** A significant difference with a “large” effect size as per Cohen’s definition.

## Data Availability

The data presented in this study are available on request from the corresponding author. The data are not publicly available due to privacy restrictions.

## Supplementary Materials

The following are available online at https://www.mdpi.com/article/10.3390/ijerph18126268/s1, Table S1: Questionnaire.

## Appendix A

**Table A1:** Breakdown of runner characteristics, preferences, and concerns by gender.

Variable	Overall Sample n (%)	19–24 n (%)	25–34 n (%)	35–44 n (%)	45–54 n (%)	55–64 (%)	65+ n (%)	Age Not Reported
**Gender**								
Man	394 (32.1)	90 (30.8)	87 (32.3)	86 (36.4)	75 (36.4)	44 (41.5)	12 (54.5)	0 (0.0)
Woman	732 (59.6)	200 (68.5)	181 (67.3)	148 (62.7)	131 (63.6)	60 (56.6)	10 (45.5)	2 (2.1)
Other	6 (0.5)	2 (0.7)	1 (0.4)	1 (0.4)	0 (0.0)	2 (1.9)	0 (0.0)	0 (0.0)
Missing	96 (7.8)	0 (0.0)	0 (0.0)	1 (0.4)	0 (0.0)	0 (0.0)	0 (0.0)	95 (97.9)
Total	1228 (100.00)	292 (100.0)	269 (100.0)	236 (100.0)	206 (100.0)	106 (100.0)	22 (100.0)	97 (100.0)
**No. of Years Running**								
0–5	408 (33.2)	183 (62.9)	111 (41.3)	60 (25.5)	41 (19.9)	13 (12.3)	0 (0.0)	0 (0.0)
6–10	304 (24.8)	77 (26.5)	91 (33.8)	62 (26.3)	44 (21.4)	26 (24.6)	3 (13.6)	1 (1.0)
11–15	130 (10.6)	9 (3.1)	41 (15.2)	40 (17.0)	22 (10.7)	11 (10.4)	6 (27.3)	1 (1.0)
16–20	87 (7.1)	1 (0.3)	11 (4.1)	35 (14.9)	25 (12.1)	13 (12.3)	2 (9.1)	0 (0.0)
21–25	34 (2.8)	0 (0.0)	6 (2.2)	10 (4.3)	12 (5.8)	5 (4.7)	1 (4.5)	0 (0.0)
26–30	31 (2.5)	0 (0.0)	0 (0.0)	6 (2.6)	15 (7.3)	8 (7.5)	2 (9.1)	0 (0.0)
30+	46 (3.7)	0 (0.0)	0 (0.0)	1 (0.4)	18 (8.7)	20 (18.8)	7 (31.8)	0 (0.0)
Missing	187 (15.2)	21 (7.2)	9 (3.3)	21 (8.9)	29 (14.1)	10 (9.4)	1 (4.5)	96 (98.0)
Total	1228 (100.0)	291 (100.0)	269 (100.0)	235 (100.0)	206 (100.0)	106 (100.0)	22 (100.0)	98 (100.00)
**Km Distance/Week**								
<10	104 (8.5)	42 (14.4)	25 (9.3)	16 (6.8)	17 (8.3)	1 (0.9)	2 (9.1)	1 (0.0)
10–20	259 (21.1)	71 (24.3)	67 (24.9)	55 (23.3)	44 (21.4)	19 (17.9)	3 (13.6)	0 (0.0)
21–30	250 (20.4)	59 (20.2)	58 (21.6)	57 (24.2)	46 (22.3)	25 (23.6)	4 (18.2)	1 (0.0)
31–40	182 (14.8)	46 (15.8)	38 (14.1)	30 (12.7)	42 (20.4)	20 (18.9)	6 27.3)	0 (0.0)
41–50	176 (14.3)	47 (12.7)	43 (16.0)	38 (16.1)	35 (17.0)	19 (17.9)	4 (18.2)	0 (0.0)
51–60	59 (4.8)	10 (3.4)	15 (5.6)	10 (4.2)	9 (4.4)	13 (12.3)	2 (9.1)	0 (0.0)
60+	103 (8.4)	27 (9.2)	238.6)	30 (12.7)	13 (6.3)	9 (8.5)	1 (4.5)	0 (0.0)
Missing	95 (7.7)	0 (0.0)	0 (0.0)	0 (0.0)	0 (0.0)	0 (0.0)	0 (0.0)	95 (97.9)
Total	1228 (100.0)	292 (100.0)	269 (100.0)	236 (100.0)	206 (100.0)	106 (100.0)	22 (100.0)	97 (100.0)
**No. of Days Running/Week**								
1	34 (2.8)	10 (3.4)	9 (3.3)	9 (3.8)	5 (2.4)	0 (0.0)	0 (0.0)	1 (0.0)
2	114 (9.3)	34 (11.6)	25 (9.3)	25 (10.6)	24 (11.7)	4 (3.8)	2 (9.1)	0 (0.0)
3	361 (29.4)	74 (25.3)	95 (35.3)	74 (31.4)	68 (33.0)	38 (35.8)	11 (50.0)	1 (0.0)
4	260 (21.2)	55 (18.8)	67 (24.9)	61 (25.8)	50 (24.3)	23 (21.7)	4 (18.2)	0 (0.0)
5	193 (15.7)	41 (14.0)	49 (18.2)	34 (14.4)	40 (19.4)	27 (25.5)	2 (9.1)	0 (0.0)
6	126 (10.3)	59 (20.2)	20 (7.4)	24 (10.2)	13 (6.3)	8 (7.5)	2 (9.1)	0 (0.0)
7	45 (3.7)	19 (6.5)	4 (1.5)	9 (3.8)	6 (2.9)	6 (5.7)	1 (4.5)	0 (0.0)
Missing	97 (7.7)	0 (0.0)	0 (0.0)	0 (0.0)	0 (0.0)	0 (0.0)	0 (0.0)	95 (97.9)
Total	1228	292 (100.0)	269 (100.0)	236 (100.0)	206 (100.0)	106 (100.0)	22 (100.0)	97 (100.0)
**Importance of Running around Others**								
1 (Not important)	438 (35.6)	105 (36.1)	100 (38.0)	100 (42.9)	78 (38.0)	39 (37.5)	7 (31.8)	9 (8.2)
2	228 (18.6)	72 (24.7)	61 (23.2)	39 (16.7)	33 (16.1)	15 (14.4)	4 (18.2)	4 (3.6)
3	275 (22.4)	65 (22.3)	66 (25.1)	56 (24.0)	46 (22.4)	26 (25.0)	8 ()36.4	8 (7.3)
4	135 (11.0)	37 (12.7)	26 (9.9)	25 (10.7)	31 (15.1)	13 (12.5)	2 (9.1)	1 (0.9)
5 (Very important)	65 (5.3)	12 (4.1)	10 (3.8)	13 (5.6)	17 (8.3)	11 (10.6)	1 (4.5)	1 (0.9)
Missing	87 (7.1)	0 (0.0)	0 (0.0)	0 (0.0)	0 (0.0)	0 (0.0)	0 (0.0)	87 (79.1)
Total	1228 (100.0)	291 (100.0)	263 (100.0)	233 (100.0)	205 (100.0)	104 (100.0)	22 (100.0)	110 (100.0)
**Prefers to Run on Sidewalk**								
Never	26 (2.1)	2 (0.7)	6 (2.2)	7 (3.0)	5 (2.4)	5 (4.7)	1 (4.5)	0 (0.0)
Rarely	129 (10.5)	39 (13.4)	19 (7.1)	26 (11.0)	24 (11.7)	13 (12.3)	7 (31.8)	1 (1.0)
Sometimes	237 (19.3)	54 (18.5)	48 (17.8)	48 (20.3)	49 (23.8)	32 (30.2)	5 (22.7)	1 (1.0)
Often	572 (46.6)	140 (47.9)	156 (58.0)	118 (50.0)	103 (50.0)	45 (42.4)	7 (31.8)	3 (3.1)
Always	171 (13.9)	57 (19.5)	40 (14.9)	37 (15.7)	24 (11.7)	11 (10.4)	1 (4.5)	1 (1.0)
Missing	93 (7.6)	0 (0.0)	0 (0.0)	0 (0.0)	1 (0.5)	0 (0.0)	1 (4.5)	91 (93.8)
Total	1228 (100.0)	292 (100.0)	269 (100.0)	236 (100.0)	206 (100.0)	106 (100.0)	22 (100.0)	97 (100.0)
**Prefers to Run on Asphalt/Paved**								
Never	10 (0.8)	3 (1.0)	1 (0.4)	5 (2.1)	1 (0.5)	0 (0.0)	0 (0.0)	0 (0.0)
Rarely	42 (3.4)	18 (6.2)	3 (1.1)	9 (3.8)	9 (4.4)	2 (1.9)	1 (4.5)	0 (0.0)
Sometimes	188 (15.3)	49 (16.8)	46 (17.1)	38 (16.1)	37 (18.0)	14 (13.2)	2 (9.1)	2 (2.1)
Often	689 (56.1)	164 (56.2)	168 (62.5)	146 (61.9)	132 (64.1)	62 (58.5)	13 (59.1)	4 (4.1)
Always	206 (16.8)	57 (19.5)	51 (19.0)	38 (16.1)	27 (13.1)	27 (25.5)	6 (27.3)	0 (0.0)
Missing	93 (7.6)	1 (0.3)	0 (0.0)	0 (0.0)	0 (0.0)	1 (0.9)	0 (0.0)	91 (93.8)
Total	1228 (100.0)	292 (100.0)	269 (100.0)	236 (100.0)	206 (100.0)	106 (100.0)	22 (100.0)	97 (100.0)
**Prefers to Run on Unpaved**								
Never	50 (4.1)	19 (6.5)	9 (3.3)	11 (4.7)	7 (3.4)	3 (2.8)	0 (0.0)	1 (1.0)
Rarely	231 (18.8)	74 (25.3)	53 (19.7)	52 (22.0)	31 (15.0)	17 (16.0)	3 (13.6)	1 (1.0)
Sometimes	453 (36.9)	106 (36.3)	112 (41.6)	85 (36.0)	87 (42.2)	51 (48.1)	9 (40.9)	3 (3.1)
Often	337 (27.4)	77 (26.4)	76 (28.3)	76 (32.2)	70 (34.0)	31 (29.2)	6 (27.3)	1 (1.0)
Always	65 (5.3)	16 (5.5)	19 (7.1)	12 (5.1)	11 (5.3)	4 (3.8)	3 (13.6)	0 (0.0)
Missing	92 (7.5)	0 (0.0)	0 (0.0)	0 (0.0)	0 (0.0)	0 (0.0)	1 (4.5)	91 (93.8)
Total	1228 (100.0)	292 (100.0)	269 (100.0)	236 (100.0)	206 (100.0)	106 (100.0)	22 (100.0)	97 (100.0)
**Prefers to Run on Track**								
Never	191 (15.6)	38 (13.0)	44 (16.4)	40 (16.9)	42 (20.4)	23 (21.7)	2 (9.1)	2 (2.1)
Rarely	383 (31.2)	88 (30.1)	79 (29.4)	87 (36.9)	80 (38.8)	35 (33.0)	12 (54.5)	2 (2.1)
Sometimes	322 (26.2)	70 (24.0)	85 (31.6)	72 (30.5)	52 (25.2)	39 (36.8)	4 (18.2)	0 (0.0)
Often	198 (16.1)	78 (26.7)	48 (17.8)	32 (13.6)	28 (13.6)	7 (6.6)	3 (13.6)	2 (2.1)
Always	41 (3.3)	18 (6.2)	13 (4.8)	5 (2.1)	4 (1.9)	1 (0.9)	0 (0.0)	0 (0.0)
Missing	93 (7.6)	0 (0.0)	0 (0.0)	0 (0.0)	0 (0.0)	1 (0.9)	1 (4.5)	91 (93.8)
Total	1228 (100.0)	292 (100.0)	269 (100.0)	236 (100.0)	206 (100.0)	106 (100.0)	22 (100.0)	97 (100.0)
**Features of the Environment Conducive to Running** (more than 1 response permitted)								
Trees along route	945 (77.0)	237 (81.2)	233 (86.6)	191 (80.9)	162 (78.6)	96 (90.6)	20 (90.9)	6 (6.2)
Access to green spaces	947 (77.1)	235 (80.5)	242 (90.0)	195 (82.6)	161 (78.2)	90 (84.9)	19 (86.4)	5 (5.2)
Public toilets	568 (46.3)	107 (36.6)	121 (45.0)	131 (55.5)	125 (60.7)	67 (63.2)	16 (72.7)	1 (1.0)
Water fountains	528 (43.0)	125 (42.8)	136 (50.6)	122 (51.7)	87 (42.2)	47 (44.3)	10 (45.5)	1 (1.0)
Access to public transit	178 (14.5)	42 (14.4)	54 (20.1)	40 (16.9)	29 (14.1)	7 (6.6)	5 (22.7)	1 (1.0)
**Concerns for Running**								
Distracted drivers	907 (73.9)	175 (59.9)	190 (70.6)	184 (78.0)	175 (85.0)	90 (84.9)	21 (95.5)	72 (74.2)
Fear of people	142 (11.6)	66 (22.6)	30 (11.2)	26 (11.0)	9 (4.4)	8 (7.5)	0 (0.0)	3 (3.1)
Both	19 (1.5)	0 (0.0)	0 (0.0)	0 (0.0)	0 (0.0)	0 (0.0)	0 (0.0)	19 (19.6)
Missing	160 (13.0)	51 (17.5)	49 (18.2)	26 (11.0)	22 (10.7)	8 (7.5)	1 (4.5)	3 (3.1)
Total	1228 (100.0)	292 (100.0)	269 (100.0)	236 (100.0)	206 (100.0)	106 (100.0)	22 (100.0)	97 (100.0)
**Importance of Avoiding Highway Pollution**								
Not at all important	86 (7.0)	21 (7.2)	20 (7.4)	19 (8.1)	13 (6.3)	12 (11.3)	1 (4.5)	0 (0.0)
Of minor importance	185 (15.1)	59 (20.2)	48 (17.8)	41 (17.4)	24 (11.7)	9 (8.5)	0 (0.0)	4 (4.1)
Somewhat important	365 (29.7)	87 (29.8)	84 (31.2)	67 (28.4)	72 (35.0)	39 (36.8)	8 (36.4)	8 (8.2)
Of major importance	258 (21.0)	56 (19.2)	64 (23.8)	58 (24.6)	47 (22.8)	23 (21.7)	5 (22.7)	5 (5.2)
Very important	254 (20.7)	68 (23.3)	53 (19.7)	51 (21.6)	49 (23.8)	21 (19.8)	7 (31.8)	5 (5.2)
Missing	80 (6.5)	1 (0.3)	0 (0.0)	0 (0.0)	1 (0.5)	2 (1.9)	1 (4.5)	75 (77.3)
Total	1228 (100.0)	292 (100.0)	269 (100.0)	236 (100.0)	206 (100.0)	106 (100.0)	22 (100.0)	97 (100.0)
**Importance of Avoiding Traffic Pollution from Arterial Streets**								
Not at all important	73 (5.9)	23 (7.9)	21 (7.8)	14 (5.9)	4 (1.9)	10 (9.4)	1 (4.5)	0 (0.0)
Of minor importance	199 (16.2)	58 (19.9)	44 (16.4)	48 (20.3)	27 (13.1)	17 (16.0)	1 (4.5)	4 (4.1)
Somewhat important	458 (37.3)	118 (40.4)	102 (37.9)	91 (38.6)	89 (43.2)	41 (38.7)	9 (40.9)	8 (8.2)
Of major importance	253 (20.6)	53 (18.2)	60 (22.3)	53 (22.5)	53 (25.7)	23 (21.7)	5 (22.7)	6 (6.2)
Very important	168 (13.7)	39 (13.4)	42 (15.6)	30 (12.7)	33 (16.0)	14 (13.2)	6 (27.3)	4 (4.1)
Missing	77 (6.3)	1 (0.3)	0 (0.0)	0 (0.0)	0 (0.0)	1 (0.9)	0 (0.0)	75 (77.3)
Total	1228 (100.0)	292 (100.0)	269 (100.0)	236 (100.0)	206 (100.0)	106 (100.0)	22 (100.0)	97 (100.0)
**Importance of Avoiding Forest Fire Pollution**								
Not at all important	271 (22.1)	56 (19.2)	60 (22.3)	66 (28.0)	54 (26.2)	26 (24.5)	6 (27.3)	3 (3.1)
Of minor importance	126 (10.3)	26 (8.9)	28 (10.4)	35 (14.8)	17 (8.3)	14 (13.2)	2 (9.1)	4 (4.1)
Somewhat important	178 (14.5)	44 (15.1)	39 (14.5)	34 (14.4)	41 (19.9)	14 (13.2)	2 (9.1)	4 (4.1)
Of major importance	222 (18.1)	65 (22.3)	53 (19.7)	39 (16.5)	40 (19.4)	19 (17.9)	3 (13.6)	3 (3.1)
Very important	354 (28.8)	101 (34.6)	87 (32.3)	62 (26.3)	54 (26.2)	33 (31.1)	9 (40.9)	8 (8.2)
Missing	77 (6.3)	0 (0.0)	2 (0.7)	0 (0.0)	0 (0.0)	0 (0.0)	0 (0.0)	75 (77.3)
Total	1228 (100.0)	292 (100.0)	269 (100.0)	236 (100.0)	206 (100.0)	106 (100.0)	22 (100.0)	97 (100.0)
**Importance of Avoiding Industry Pollution**								
Not at all important	132 (10.7)	38 (13.0)	24 (8.9)	29 (12.3)	22 (10.7)	16 (15.1)	2 (9.1)	1 (1.0)
Of minor importance	216 (17.6)	58 (19.9)	63 (23.4)	51 (21.6)	30 (14.6)	11 (10.4)	1 (4.5)	2 (2.1)
Somewhat important	337 (27.4)	81 (27.7)	72 (26.8)	70 (29.7)	70 (34.0)	27 (25.5)	5 (22.7)	12 (12.4)
Of major importance	258 (21.0)	60 (20.5)	56 (20.8)	62 (26.3)	49 (23.8)	23 (21.7)	5 (22.7)	3 (3.1)
Very important	203 (16.5)	55 (18.8)	51 (19.0)	24 (10.2)	35 (17.0)	27 (25.5)	7 (31.8)	4 (4.1)
Missing	82 (6.7)	0 (0.0)	3 (1.1)	0 (0.0)	0 (0.0)	2 (1.9)	2 (9.1)	75 (77.3)
Total	1228 (100.0)	292 (100.0)	269 (100.0)	236 (100.0)	206 (100.0)	106 (100.0)	22 (100.0)	97 (100.0)
