# Supplementary material for: Environmental Preferences and Concerns of Recreational Road Runners
Source: Int J Environ Res Public Health. 2021 Jun 10;18(12):6268. doi: 10.3390/ijerph18126268 (PMC8296135; doi:10.3390/ijerph18126268)
Supplement: Supplementary file 1 [file ijerph-18-06268-s001.zip › ijerph-1203687-supplementary.pdf]

International Journal of
Environmental Research and Public Health

MDPI

Article

Environmental Preferences and Concerns of Recreational Road Runners

Nadine Schuurman [1,*], Leah Rosenkrantz [1] and Scott A. Lear [2]

[1] Faculty of Environment, Simon Fraser University, 8888 University Drive, Burnaby, BC V5A 1S6, Canada; lrosenkr@sfu.ca
[2] Faculty of Health Sciences, Simon Fraser University, 8888 University Drive, Burnaby, BC V5A 1S6, Canada; salear@sfu.ca
* Correspondence: nadine@sfu.ca

Citation: Schuurman, N.; Rosenkrantz, L.; Lear, S.A. Environmental Preferences and Concerns of Recreational Road Runners. *Int. J. Environ. Res. Public Health* **2021**, *18*, 6268. https://doi.org/10.3390/ijerph18126268

Academic Editor: Paul B. Tchounwou

Received: 15 April 2021
Accepted: 4 June 2021
Published: 10 June 2021

Publisher's Note: MDPI stays neutral with regard to jurisdictional claims in published maps and institutional affiliations.

Copyright: © 2021 by the authors. Submitted for possible open access publication under the terms and conditions of the Creative Commons Attribution (CC BY) license (http://creativecommons.org/licenses/by/4.0/).

Supplementary Material

Table S1. Questionnaire.

No.	Question
**1**	How important is it for you to run around/with others? (scale from 1 to 5, with 1 being not important and 5 being very important)
**2**	Safety wise, what are your primary concerns while running? (Check all that apply)<br>a. Fear of people<br>b. Distracted/aggressive drivers<br>c. Other (please specify)
**3**	How important is avoiding pollution from each of the following sources? (options included: ‘not at all important’, ‘of minor importance’, ‘somewhat important’, ‘of major importance’, ‘very important’)<br>a. Highways<br>b. Traffic on arterial streets<br>c. Forest fires<br>d. Industry<br>e. Other (please specify)
**4**	How frequently do you seek out each of the following types of running surfaces? (options included: ‘never’, ‘rarely’, ‘sometimes’, ‘often’, ‘always’)<br>a. Sidewalks<br>b. Asphalt/paved paths<br>c. Unpaved paths<br>d. Running tracks
**5**	What features of the urban environment are conducive to running? (Check all that apply)<br>a. Trees along running routes<br>b. Access to green spaces/parks<br>c. Public toilets<br>d. Water fountains<br>e. Access to public transit<br>f. Other (please specify)
**6**	What is your age?<br>a. Under 18<br>b. 18-24<br>c. 25-34<br>d. 35-44<br>e. 45-54<br>f. 55-64<br>g. 65+
**7**	How many years have you been running?
**8**	Approximately how many kilometers do you run a week?<br>a. Less than 10<br>b. 10-20<br>c. 20-30<br>d. 30-40

	e. 40-50<br>f. 50-60<br>g. 60+
**9**	How many days a week do you run?<br>a. One<br>b. Two<br>c. Three<br>d. Four<br>e. Five<br>f. Six<br>g. Seven
**10**	What is your gender?<br>a. Woman<br>b. Man<br>c. Other
